# Development of the Pharmacist’s Stress Scale for Home Care (PSS) and evaluation of its reliability and validity

**DOI:** 10.1186/s40545-023-00610-8

**Published:** 2023-12-28

**Authors:** Ayana Kani, Manako Hanya, Hiroyuki Kamei, Kenta Murotani

**Affiliations:** 1 Laboratory of Clinical Pharmacy Practice and Heath Care Management, Faculty of Pharmacy, Meijo University, 150 Yagoyama, Tenpaku, Nagoya, Japan; 2 Biostatistics Center, Kurume University, 67 Asahi-Machi, Kurume, Fukuoka Japan

**Keywords:** Home care, Pharmacist, Stress, Insurance-covered pharmacy, Questionnaire, Interpersonal work

## Abstract

**Background:**

As the needs for home care increase, contact with patients and jobs out of the pharmacy such as the patients’ homes have increased, and there is concern that the situation increases pharmacists’ work-related stress. Stress deteriorates pharmacists’ quality of life (QOL) and affects the quality of services they provide. In this study, we developed a scale for the measurement of stress of pharmacists engaged in home care and evaluated it in pharmacists in 3 prefectures of the Tokai district, Japan.

**Methods:**

Based on the stress factors of pharmacists in home care extracted by previous studies, a 59-item questionnaire was developed. The questionnaires were sent to 1785 pharmacies engaged in home care in 3 prefectures of the Tokai district, and anonymous responses were obtained from 399 (valid response rate: 22.4%). The answers to each question were scored using 5-point scale (1: I feel no stress to 5: I always feel strong stress).

**Results:**

As a result of factor analysis, the Pharmacist’s Stress Scale for Home Care (PSS) was prepared with 51 items, i.e., 14 related to the first factor, “difficulty and feeling of incompetence in patient care”, 9 related to the second factor, “relationship with superiors and work environment”, 13 related to the third factor, “burdens related to work load and work contents”, 10 related to the fourth factor, “communication with patients and families”, and 5 related to the fifth factor, “communication with other professions”. Cronbach’s α coefficients for the entire scale and each factor were ≥ 0.833, and sufficient internal consistency was obtained.

**Conclusions:**

The scale developed in this study is considered to be useful for the measurement of stress of pharmacists derived from interpersonal work and home care services. Leaving the job and burnout are expected to be prevented by perceiving the stress level of the pharmacists by themselves using this scale and coping with stress.

**Supplementary Information:**

The online version contains supplementary material available at 10.1186/s40545-023-00610-8.

## Background

The rate of aging of the Japanese population is highest in the world and is expected to remain high in the future [1]. Under the circumstances of Japan being a super aging society, the Ministry of Health, Labour and Welfare is promoting the development of community-based integrated care systems to help people continue to live according to their wishes in their familiar local community [2]. Pharmacies providing home care services account for more than 40% of all insurance-covered pharmacies in Japan, and their number is increasing every year [3, 4]. The tasks of pharmacists in home care include checking of adherence, proposing prescriptions appropriate for each patient’s lifestyle, support for patients with poor medication adherence, and early detection of adverse reactions by checking vital signs [5]. To provide safe drug therapy, pharmacists are expected to increase contact with patients and strengthen cooperation with other professions [6]. At the same time, higher levels of expertise and communication ability are required for the pharmacists’ jobs from the viewpoint of promotion of interprofessional work, and pharmacists’ jobs are expected to shift from the conventional object-centered tasks such as drug dispensing to interpersonal-oriented tasks involving face-to-face contact with patients and other healthcare workers [7]. According to the Vision of “Pharmacy for Patients” [7] formulated by the Ministry of Health, Labour, and Welfare in 2015, pharmacists’ interpersonal work is expanding, as medication guidance not only at the time of dispensing, but also after dispensing and continuous monitoring of medication status became mandatory in 2022. [8]

Healthcare workers including pharmacists are under occupational stress due to a wide variety of factors, such as excessive workload [9], increasing the responsibility to patients [10, 11], lack of required skills [11], lack of rewards for efforts [12], and subpar work environments [9]. In 2008, 83.6% of the Japanese pharmacists providing services covered by health insurance felt occupational stress [13], but pharmacists’ workload is expected to increase with a further increase in stress against a backdrop of the demand for home care associated with population aging and cooperation with other professions and shift from objective to interpersonal work. David et al. [14] showed that the occupational stress of pharmacy pharmacists was related to mental/physical problems, such as anxiety, depression, fatigue, and sleep disorders. In addition, Yasser [15] observed that the occupational stress of pharmacists reduces their own QOL and leads to a decline in the quality of services they provide. It is concerned that these stressors induce an increase in the turnover rate [9] make it difficult to cope with the growing needs for home care. The management of stress of pharmacists has emerged as an important issue for providing high-quality healthcare.

Sally et al. [16] in the UK investigated the relationships of occupational stress felt by pharmacists with the characteristics of individual pharmacists, task contents, and characteristics of the organization they belonged to and reported that pharmacists at local pharmacies are more likely to be exposed to various occupational stressors including work–life balance and overwork. Recently, studies on the stress of pharmacists have also begun in Japan, but they are still fewer than in foreign countries. In addition, while studies on the relationships of stress primarily due to objective work with QOL and job satisfaction of pharmacists have also been conducted [17], research on stressors caused by interpersonal work has been insufficient. In addition, there has been no study on pharmacists’ stress focusing on the scenes of home care. The questionnaire used in the previous study [18] was designed for nurses, and stress items specific to pharmacists were insufficient. To cope with pharmacists’ stress in the changing circumstances of their work, a simple questionnaire designed to identify factors involved as stressors is needed. In the previous study [18], stress items other than the items of the questionnaire were also identified from pharmacists’ free comments. Based on the stressors of pharmacists in home care services extracted in the previous study [18], including these items, we developed a new questionnaire, the Pharmacist’s Stress Scale for Home Care (PSS), clarified the factor structure, and examined the reliability in the present study.

## Methods

### Study design

This is a qualitative cross-sectional study using a 5-point scale questionnaire.

### Setting

This study was carried out from April to September 2022. To enrolled pharmacies, “Questionnaire on Home Pharmaceutical Service”, “PSS”, and a Japanese version of “Effort–Reward Imbalance (ERI) Questionnaire (see Additional file 1)” [19] were mailed. This study was carried out with approval (R1–8) by the Meijo University Ethical Review Board in compliance with the Ethical Guidelines for Medical and Health Research Involving Human Subjects.

### Participants recruitment

A search was made for pharmacies providing insured home care services on the websites of the Aich Pharmaceutical Association, Gifu Pharmaceutical Association, and Mie Pharmaceutical Association, and 623, 455, and 707 pharmacies, respectively, with a total of 1785 pharmacies were enrolled. The 3 prefectures of the Tokai district targeted in this study are the most universal regions in Japan in terms of medical care. Therefore, we expanded the target area by adding insurance-covered pharmacies in Gifu and Mie prefectures to those in Aichi Prefecture examined in the previous study [18] to make it possible to create a more generalized questionnaire.

### Survey development

The “Questionnaire on Home Pharmaceutical Service” consists of 9 items: the pharmacist’s sex, age, duration of career, engagement in home care, current place and type of employment, weekly waged working hours, academic background, and whether the pharmacist had the experience of home care of terminal patients. The questions were answered from 3 choices: “Yes”, “No”, and “Others”.

In a previous study conducted in pharmacists working at pharmacies providing insured home pharmaceutical services in Aichi Prefecture [18], 43 and 33 stressors related to 7 factors were extracted from the answers to the PSS and free comments, respectively. In the present study, 59 items were selected from the 82 items extracted in the previous study [18] by carefully evaluating their contents to develop a questionnaire about home pharmaceutical services. Furthermore, following the method used by Makita [20], 3 questions related to “social-desirability bias” were added. “Social-desirability bias” is “the response or tendency of survey responders to distort their answers in a manner more socially desirable under the influence of the judgment of society about whether the matter in question is desirable or not” [21]. To examine the effect of this tendency, we added 3 items (No.20: “I do not have meals at home in such a good manner as when I eat out”, No.43: “I do not like everyone that I know (my acquaintances)”, and No. 59: I would not mind being schizophrenic at all should I develop the disease”, and reconstituted the PSS with a total of 62 questions. Each question was responded using a 5-point scale: 5: “I always feel strong stress.”, 4: “I always feel stress.”, 3: “I occasionally feel stress.”, 2: “I feel little stress.”, and 1: “I feel no stress.” Before mailing the questionnaire to the subjects, the author, a university faculty member specializing in psychiatry, and a university faculty member specializing in medical communication checked each item of the questionnaire.

### Statistical analysis

Statistical analysis was performed using IBM SPSS Statistics 28.0 (IBM Japan, Tokyo, Japan), with the significance level set at *p* < 0.05.

Prior to factor analysis, the mean score and standard deviation were calculated, and the ceiling and floor effects were confirmed for each item. Next, to evaluate the goodness of fit of the number of factors, Kaiser–Mayer–Olkin’s sample validity measure and Bartlett’s sphericity test were conducted. To clarify the factor structure from the 51 items, factor analysis was performed using the generalized least-squares method for factor extraction and promax rotation. The number of factors was determined based on the scree test and interpretability of the factors. Items with a factor loading of 0.35 or higher for a single factor were considered meaningful as items constituting each factor, and were assigned to a specific factor. The name that best characterizes the group of items assigned to each specific factor was examined and given to that factor. Cronbach’s α coefficient was calculated to evaluate the internal consistency of the entire scale and among the subscales of each factor.

Also, to evaluate the criterion-related validity of the PSS of the 51 items extracted by factor analysis (see Additional file 2), data were collected using ERI, and their correlations with the PSS were confirmed. ERI, which consists of 3 factors, i.e., “effort”, “reward”, and “overcommitment”, is a scale used to evaluate the work environment by assuming it as stressful when it is under a high burden-low reward condition in which the effort spent in working life is not balanced with the reward obtained or expected to be obtained from it. Its validity is already recognized [19]. In the present study, Tsutsumi’s Japanese version of the ERI [19] prepared with permission by Siegrist [22] was used.

Furthermore, to examine the effect of “social-desirability bias” on the PSS trimmed to 51 items as a result of factor analysis, the correlation between the total score of the 3 items related to “social-desirability bias” and the total score of the 51-item PSS was examined.

## Results

### Sample inclusion and exclusion criteria

Of the 1785 pharmacies surveyed, anonymous responses were obtained from 491 (response rate: 27.5%). The number of data was examined after excluding 3 questions related to “social-desirability bias” from the 62 questions adopted in the PSS used in the present study. A total of 4 pages were spent on the “Questionnaire on Home Pharmaceutical Services” and PSS, but responses from 51 pharmacies answered none of the questions on these pages by an oversight of the pages. There were one or more omissions of other data in the responses from 41 pharmacies. Therefore, a total of 92 responses with even a single defect in the data were excluded by the listwise method, and 399 responses with complete data were analyzed (valid response rate: 22.4%).

### Subjects’ attributes and background

The 399 subjects consisted of 244 males (61.2%) and 155 females (38.8%) with a mean age of 45.2 ± 11.8 years. The mean duration of career was 21.1 ± 12.4 years, mean duration of engagement in home care was 6.5 ± 5.7 years, and mean weekly waged working hours was 42.8 ± 14.2 h. The current place of work was most often a health insurance pharmacy (386, 96.7%), and the current employment type was manager in 177 (44.4%) and full-time employee in 143 (35.8%), which accounted for about 80% of all respondents. The academic background was most often 4-year college of pharmacy (269, 67.4%). The experience of giving care to terminal patients in home care was present in 231 (57.9%) and absent in 165 (41.4%) (Table 1).

**Table 1:** Subjects’ attributes and background (*n* = 399)

Item	*n*	%
Sex		
Male	244	61.2
Female	155	38.8
Age		
20–29	33	8.3
30–39	116	29.1
40–49	109	27.3
50–59	92	23.1
60–69	39	9.8
70–79	9	2.3
80–89	1	0.3
Duration of career (from obtaining license to the present)		
< 10 years	84	21.1
10–19 years	109	27.3
20–29 years	96	24.1
30–39 years	77	19.3
40–49 years	26	6.5
≥ 50 years	7	1.8
Duration of engagement in home care		
< 5 years	179	44.9
5–9 years	131	32.8
10–14 years	57	14.3
15–19 years	13	3.3
20–24 years	12	3.0
≥ 25 years	7	1.8
Current place of work		
Health insurance pharmacy	386	96.7
Drug store	12	3.0
Others	1	0.3
Current employment type		
Owner	67	16.8
Manager	177	44.4
Full-time employee	143	35.8
Part-time employee	11	2.8
Others	1	0.3
Weekly waged working hours		
< 20 h	7	1.8
20–39 h	43	10.8
40–59 h	329	82.5
60–79 h	15	3.8
80–99 h	1	0.3
≥ 100 h	4	1.0
Academic background		
4-year college of pharmacy	269	67.4
4-year college of pharmacy + graduate course	40	10
6-year college of pharmacy	90	22.6
6-year college of pharmacy + graduate course	0	0
Experience of giving care to terminal patients in home care		
Yes	231	57.9
No	165	41.4
Others	3	0.8

### Clarification of factor structure and evaluation of reliability

Means and standard deviations were calculated concerning 59 of the 62 items adopted in the PSS by excluding the 3 questions concerning “social-desirability bias”. Since no ceiling or floor effect was observed in any of the items (Table 2), the initial factor analysis was performed concerning all 59 items (factor extraction by generalized least-squares method, promax rotation). The number of factors was estimated using the scree test and decided to be 5 in consideration of the possibility of factor interpretation. The Kaiser–Mayer–Olkin measure of sample adequacy was 0.955, the result of Bartlett’s test of sphericity was *p* < 0.01, showing a significant difference compared with the identity matrix, and the validity of implementation of factor analysis was confirmed. Next, by assuming the number of factors as 5, the second factor analysis was performed (factor extraction by generalized least-squares method, promax rotation). As a result, 2 items with a factor loading of < 0.35 and 4 items with a factor loading of ≥ 0.35 across multiple factors were excluded, and the third factor analysis was performed again by assuming 5 factors (factor extraction by generalized least-squares method, promax rotation). As a result, the content of the statement No. 50 “I feel my lack of knowledge when I work with other professions,” was considered to be inconsistent with other items included in the first factor. Since the values of Cronbach’s α coefficient calculated by including and excluding No. 50 were comparable, No. 50 was excluded from the first factor. Regarding the second factor, the contents of No. 30 “There are staff members whom I would not like to work with,” and No. 54 “I have to work with uncooperative staff members,” were similar. The Cronbach’ α coefficient was calculated by excluding either item at a time, and since a high value was obtained when No. 30 was excluded, it was eliminated from the second factor. Table 3 shows the final factor pattern and inter-factor correlations after promax rotation. All subscales showed positive correlations. The inter-factor correlation between the fourth and fifth factors was 0.607 (Table 3, bottom), and a stronger positive correlation between these factors than among other factors was confirmed.

**Table 2:** Descriptive statistics of questions, ceiling effect, floor effect (*n* = 399)

		Mean	Standard deviation	Ceiling effect	Floor effect
	1. I cannot have prospects of patient care as a pharmacist	2.88	0.900	3.78	1.98
	2. I cannot give satisfactory care to patients	3.04	0.821	3.86	2.22
	3. I cannot have contact with or talk to patients at leisure	2.86	0.962	3.83	1.90
*	4. My superiors do not understand my feelings*2	2.68	1.242	3.93	1.44
	5. My superiors have views different from mine	2.73	1.251	3.98	1.48
	6. There is too much to do other than pharmacist’s work, such as office work	3.11	1.157	4.27	1.95
	7. I cannot secure enough time to rest	3.12	1.183	4.31	1.94
	8. I cannot agree with the physicians’ policies or thoughts	2.60	0.948	3.55	1.66
*	9. I have to give care to patients who refuse me*2	3.26	1.050	4.31	2.21
	10. The poor hygienic environment of the homes I visit makes me feel disagreeable	2.98	1.145	4.13	1.84
	11. I cannot intervene in home care as I wish to and exercise my professional skill	2.86	0.971	3.83	1.89
*	12. The relations among workers of different professions are poor*2	2.76	1.063	3.82	1.69
	13. I cannot give support to suffering patients or families	2.99	0.968	3.96	2.02
	14. I have to see patients unable to have prospects for their future	2.86	0.960	3.82	1.90
	15. Patients shout at or talk abusively to me	3.24	1.286	4.52	1.95
	16. My superiors do not trust me	2.57	1.238	3.81	1.33
	17. I have no one at my workplace to confide in or consult with	2.62	1.201	3.82	1.42
	18. I do not have enough time to give satisfactory care	2.87	0.952	3.82	1.92
	19. I have difficulty in arranging time for home visits	3.24	1.030	4.27	2.21
	21. I am not sure about what explanation patients and families are given by the physicians about the treatment and prognosis	2.88	0.855	3.74	2.03
	22. Care that I gave for the good of patients and families is misunderstood by them	3.04	1.013	4.05	2.02
*	23. I have trouble with parking spaces and traffic jams when I visit homes by car*1	2.89	1.156	4.04	1.73
	24. I am paid inadequately for my drug dispensation workload	3.12	1.138	4.26	1.98
	25. The work of pharmacists is not understood by other professions	2.95	1.033	3.98	1.92
	26. I am helpless in the care of terminal patients	2.96	1.063	4.03	1.90
	27. I am not sure how I should deal with patients not informed of their prognoses	2.88	1.053	3.93	1.82
	28. Patients order me to do things	2.77	1.184	3.96	1.59
	29. My superiors respond slowly	2.74	1.248	3.99	1.50
*	30. There are staff members whom I would not like to work with*3	2.74	1.322	4.06	1.42
	31. I am required to respond immediately in emergencies	3.10	1.121	4.22	1.97
	32. Physicians respond slowly	2.66	0.995	3.65	1.66
	33.I must give care to patients who change their language and attitude according to the profession of the healthcare worker	2.54	1.009	3.55	1.53
	34. I am not trusted by patients and families	2.75	1.104	3.85	1.64
	35. The employer’s policy prevents me from doing what I want to do for patients	2.54	1.173	3.71	1.36
	36. Preparation of papers such as reports complicates my job	3.41	1.083	4.49	2.33
	37. The services that I can provide with my skills or by my pharmacy are deficient	2.95	0.968	3.92	1.98
	38. I cannot adequately handle patients’ and families’ anxiety and wishes	2.92	0.934	3.85	1.99
	39. Death of patients I am in charge of or I have made friends with	3.06	1.135	4.19	1.93
	40. Patients commit harassing and malicious behavior	2.95	1.247	4.20	1.71
	41. My colleagues and workers of other professions do not help me when I am in trouble	2.89	1.165	4.05	1.72
	42. Manpower is deficient	3.41	1.094	4.50	2.31
	44. I have to handle unexpected jobs	3.14	0.978	4.11	2.16
	45. My work is not understood by physicians	2.67	0.917	3.59	1.75
	46. I have to take care of patients who change their language and attitude according to the pharmacist providing service	2.62	0.985	3.60	1.63
	47. My sincere care is not understood by patients and families	2.69	0.963	3.65	1.73
	48. I am urged by the management to improve work efficiency	2.83	1.276	4.11	1.55
	49. I feel burdened by the heavy liaison work with other profession	2.82	1.000	3.82	1.82
*	50. I feel my lack of knowledge when I work with other professions*3	3.14	0.952	4.10	2.19
	51. I am helpless about the exacerbation of patients’ symptoms	2.92	0.964	3.89	1.96
*	52. I see patients treating their families harshly or the other way around*2	2.85	1.001	3.85	1.85
	53. My superiors do not support me when I am in trouble	2.74	1.190	3.93	1.55
	54. I have to work with uncooperative staff members	3.09	1.198	4.28	1.89
	55. I have to work under time pressure	3.49	1.079	4.57	2.41
	56. I have to deal with patients outside duty hours	3.22	1.135	4.36	2.09
	57. I have to work with uncooperative physicians	2.96	1.139	4.10	1.82
	58. I have to give care to patients difficult to have communication with	2.99	1.017	4.01	1.98
	60. I feel burdened by having to visit homes alone (e.g., carrying a heavy baggage, fear of entering the house of a man living alone)	2.54	1.206	3.75	1.33
	61. I cannot give care to all patients equally when I have to take care of many home visits	2.59	0.975	3.57	1.62
*	62. It is difficult to smoothly share information with workers of other professions*1	2.81	0.951	3.76	1.86

*Excluded items *1: Factor loading < 0.35 *2: Factor loading ≥ 0.35 in multiple factors *3: Excluded after evaluating content validity of contents

**Table 3:** Results of factor analysis of the PSS

The contents of items	Factor				
	1	2	3	4	5
1st factor: Difficulty and feeling of incompetence in patient care					
13. I cannot give support to suffering patients or families	0.841	− 0.020	− 0.092	0.113	− 0.046
14. I have to see patients unable to have prospects for their future	0.813	0.018	− 0.076	0.124	− 0.115
2. I cannot give satisfactory care to patients	0.802	0.147	− 0.065	− 0.281	0.097
3. I cannot have contact with or talk to patients at leisure	0.732	0.199	0.105	− 0.281	− 0.100
51. I am helpless about the exacerbation of patients’ symptoms	0.726	− 0.138	0.154	0.037	− 0.005
38. I cannot adequately handle patients’ and families’ anxiety and wishes	0.702	0.125	− 0.005	0.010	0.028
39. Death of patients I am in charge of or I have made friends with	0.681	− 0.125	0.045	0.277	− 0.219
26. I am helpless in the care of terminal patients	0.642	− 0.245	0.022	0.137	0.182
1. I cannot have prospects of patient care as a pharmacist	0.640	0.023	− 0.064	− 0.093	0.167
27. I am not sure how I should deal with patients not informed of their prognoses	0.625	− 0.118	− 0.004	0.341	− 0.069
11. I cannot intervene in home care as I wish to and exercise my professional skill	0.549	− 0.038	− 0.065	0.006	0.277
37. The services that I can provide with my skills or by my pharmacy are deficient	0.544	0.085	0.139	− 0.155	0.220
18. I do not have enough time to give satisfactory care	0.517	0.307	0.206	− 0.243	0.052
21. I am not sure about what explanation patients and families are given by the physicians about the treatment and prognosis	0.449	− 0.102	0.015	0.187	0.213
2nd factor: Relationship with superiors and work environment					
53. My superiors do not support me when I am in trouble	− 0.019	0.794	0.109	0.030	− 0.025
5. My superiors have views different from mine	0.009	0.788	0.024	− 0.008	− 0.035
29. My superiors respond slowly	− 0.076	0.758	− 0.013	0.112	0.046
54. I have to work with uncooperative staff members	− 0.171	0.702	0.158	0.109	0.053
16. My superiors do not trust me	0.099	0.701	− 0.251	0.246	0.014
17. I have no one at my workplace to confide in or consult with	0.099	0.687	− 0.070	0.034	0.012
35. The employer’s policy prevents me from doing what I want to do for patients	0.158	0.590	− 0.130	0.168	0.094
41. My colleagues and workers of other professions do not help me when I am in trouble	0.074	0.534	0.112	0.312	− 0.114
48. I am urged by the management to improve work efficiency	0.084	0.527	0.221	0.108	− 0.109
3rd factor: Burdens related to work load and work contents					
44. I have to handle unexpected jobs	− 0.094	− 0.181	0.820	0.115	0.044
55. I have to work under time pressure	0.007	0.209	0.742	− 0.147	0.019
56. I have to deal with patients outside duty hours	− 0.194	− 0.030	0.720	0.270	− 0.063
36. Preparation of papers such as reports complicates my job	0.058	− 0.022	0.596	− 0.097	0.058
31. I am required to respond immediately in emergencies	0.090	− 0.172	0.591	0.335	− 0.137
7. I cannot secure enough time to rest	0.035	0.209	0.562	− 0.208	− 0.052
19. I have difficulty in arranging time for home visits	0.195	0.011	0.542	− 0.027	0.042
60. I feel burdened by having to visit homes alone (e.g., carrying a heavy baggage, fear of entering the house of a man living alone)	0.147	− 0.048	0.517	0.140	− 0.094
49. I feel burdened by the heavy liaison work with other profession	0.039	0.014	0.513	0.069	0.173
42. Manpower is deficient	0.067	0.316	0.497	0.026	− 0.083
6. There is too much to do other than pharmacist’s work, such as office work	0.085	0.082	0.454	− 0.181	0.099
24. I am paid inadequately for my drug dispensation workload	− 0.037	0.087	0.421	0.046	0.158
61. I cannot give care to all patients equally when I have to take care of many home visits	0.304	− 0.069	0.420	0.098	0.027
4th factor: communication with patients and families					
28. Patients order me to do things	− 0.153	0.138	0.080	0.758	− 0.017
15. Patients shout at or talk abusively to me	0.016	0.229	− 0.053	0.735	− 0.116
40. Patients commit harassing and malicious behavior	− 0.051	0.331	− 0.118	0.724	− 0.071
33.I must give care to patients who change their language and attitude according to the profession of the healthcare worker	0.014	0.040	0.131	0.576	0.139
34. I am not trusted by patients and families	0.182	0.301	− 0.214	0.526	0.115
46. I have to take care of patients who change their language and attitude according to the pharmacist providing service	− 0.132	− 0.021	0.153	0.516	0.381
22. Care that I gave for the good of patients and families is misunderstood by them	0.325	0.129	− 0.177	0.512	0.050
10. The poor hygienic environment of the homes I visit makes me feel disagreeable	0.019	0.023	0.222	0.473	− 0.011
47. My sincere care is not understood by patients and families	0.157	0.077	− 0.043	0.454	0.303
58. I have to give care to patients difficult to have communication with	0.108	0.006	0.261	0.418	0.042
5th factor: communication with other professions					
45. My work is not understood by physicians	− 0.042	− 0.003	0.042	0.045	0.916
8. I cannot agree with the physicians’ policies or thoughts	0.177	0.063	− 0.082	− 0.023	0.559
25. The work of pharmacists is not understood by other professions	0.193	− 0.142	0.104	0.161	0.501
57. I have to work with uncooperative physicians	− 0.151	0.229	0.108	0.296	0.392
32. Physicians respond slowly	− 0.015	0.238	0.082	0.153	0.338
Inter-factor correlations	1	2	3	4	5
1	–	0.545	0.529	0.532	0.590
2	0.545	–	0.461	0.552	0.543
3	0.529	0.461	–	0.490	0.558
4	0.532	0.552	0.490	–	0.607
5	0.590	0.543	0.558	0.607	–

The first factor consisted of 14 items, the factor loading value was particularly high in No. 13 “I cannot give support to suffering patients or families,” No. 14 “I have to see patients unable to have prospects for their future,” and No. 2 “I cannot give satisfactory care to patients,” and the factor was named “difficulty and feeling of incompetence in patient care”. The second factor consisted of 9 items. The factor loading value was particularly high in No. 53 “My superiors do not support me when I am in trouble”, No. 5 “My superiors have views different from mine”, and No. 29 “My superiors respond slowly,” and this factor was named “relationship with superiors and work environment” in consideration also of the contents of other items. The third factor consisted of 13 items. The factor loading value was particularly high in No. 44 “I have to handle unexpected jobs,” No. 55 “I have to work under time pressure,” and No. 56 “I have to deal with patients outside duty hours,” and the factor was named “burdens related to work load and work contents”. The fourth factor consisted of 10 items. The factor loading value was particularly high in No. 28 “Patients order me to do things,” No. 15 “Patients shout at or talk abusively to me,” and No. 40 “Patients commit harassing and malicious behavior,” and this factor was named “communication with patients and families” in consideration also of the contents of other items. The fifth factor consisted of 5 items. The factor loading value was particularly high in No. 45 “My work is not understood by physicians,” and this factor was named “communication with other professions” in consideration of the contents of other items.

To examine the internal consistency, Cronbach’s α coefficient was calculated for the entire scale and each factor, and sufficient internal consistency could be confirmed with α = 0.968 for the entire scale and α = 0.833–0.932 for various factors.

Criterion-related validity was evaluated using the correlation coefficient between the total score of each factor and total score of the entire scale of the PSS and the total score of each factor and total score of the entire scale of the conventional ERI questionnaire in the 399 subjects (Table 4). Since there were defects in the data collected by ERI in 45 of the 399 subjects, analysis was performed concerning the 354 subjects with complete data after excluding the 45 subjects. As a result, significant positive correlations (*r* = 0.116–0.540, *p* < 0.05) were observed except between the total score of the fifth factor “communication with other professions” of the PSS and the total score of “overcommitment” of the ERI scale (*r* = 0.07, *p* = 0.188), and the PSS was confirmed to have criterion-related validity.

**Table 4:** Evaluation of criterion-related validity (correlation coefficients among factors)

		Factors used in the Japanese version of ERI (effort–reward imbalance model) questionnaire			
		Total score of effort	Total score of reward	Total score of overcommitment	Total score of ERI
Factors of PSS	Total score of 1st factor	0.272**	0.278**	0.274**	0.333**
	Total score of 2nd factor	0.168**	0.368**	0.153**	0.316**
	Total score of 3rd factor	0.553**	0.396**	0.401**	0.540**
	Total score of 4th factor	0.120*	0.148**	0.116*	0.160**
	Total score of 5th factor	0.153**	0.218**	0.070	0.203**
	Total score of all 51 items	0.322**	0.346**	0.267**	0.389**

**p < 0.01, *p < 0.05

### Social-desirability bias

The total score of the entire scale of the PSS was not correlated with the total score of the 3 items for the evaluation of social desirability in the 399 subjects (*r* = − 0.154, *p* = 0.002). The PSS was confirmed not to be affected by “social-desirability bias”.

## Discussion

### Characteristics of stress among pharmacists in home care

Stress of pharmacists outside the pharmacy is increasing in addition to the stress of jobs in the pharmacy due to the increase in home care services and shift from objective work to interpersonal work. To prevent the decline in QOL of pharmacists and increase in the employment separation rate and to cater to the needs of home pharmaceutical services, clarification of factors that work as stressors is necessary. In this study, stress of pharmacists related to home pharmaceutical services was investigated in pharmacists at pharmacies providing home pharmaceutical services under health insurance in 3 Tokai prefectures, and the factor structure and validity of the PSS were evaluated.

Fifty-one items were selected based on factor analysis, Cronbach’s α coefficient, and correlation coefficient concerning all 59 items of the PSS. Regarding the reliability of the results of factor analysis, Cronbach’s α coefficients of the entire scale and each factor was ≥ 0.833, and sufficient internal consistency was confirmed. As a result, the PSS was prepared with a total of 51 items consisting of 14 items of the first factor “difficulty and feeling of incompetence in patient care”, 9 items of the second factor “relationship with superiors and work environment”, 13 items of the third factor “burdens related to work load and work contents”, 10 items of the fourth factor “communication with patients and families”, and 5 items of the fifth factor “communication with other professions”.

The first factor “difficulty and feeling of incompetence in patient care” consisted of items of stress derived from the current state of not being able to provide high-quality care to patients because of the respondent’s immaturity as a pharmacist and inability to fully exercise his/her professional ability. A study in nurses [23] reported that the subjects felt a sense of incompetence or guilt when they considered that the gap between the ideal and reality of patient care was derived from the subject’s own constitution or personality or when they perceived inadequacy of patient care, leading to burnout. The results concerning the first factor were the same, and pharmacists are considered to be feeling similar stress. In addition, the first factor included an item of stress related to patients’ death, No. 39 “death of patients I am in charge of or I have made friends with”. Conventional services of pharmacists were mostly completed in the pharmacy, and they rarely had direct involvement in patients’ death. Recently, however, as pharmacists began to participate in home care, opportunities in which pharmacists intervene in the patients’ living environments have increased. According to the study by Harada et al. in nurses [24], among the occupational stressors of nurses, direct experience of patients’ death and remorse over the inability to prevent it caused the greatest stress. For pharmacists, direct experience of patients’ death and the sense of incompetence in terminal care were also suggested to be stressors.

It is also important for individual pharmacists to establish good relations with their superiors and colleagues to provide patients with safe medical treatment. The second factor “relationship with superiors and work environment” included stress items related to the relationship with superiors and colleagues at the workplace. In a study involving hospital healthcare workers including pharmacists [25], inadequate support by superiors and working with uncooperative colleagues were work-related stressors, and the results of the present study support this view. The second factor is considered to be stress caused by the difficulty in coping with work-related problems due to inadequate cooperation among staff members at the workplace.

To promote interpersonal work, which are presently considered important, smooth execution of objective work is a prerequisite. The third factor “burdens related to work load and work contents” included stress items related to the heaviness of work load expected of pharmacists and the lack of time to meet the requirements. There have been a number of studies that suggested that overloading of pharmacists and manpower shortage cause stress and lead to increases in the development of burnout and employment separation rate and a decline in job satisfaction [9, 17], and the results of the present study were in agreement. Pharmacists are engaged in a wide variety of tasks including not only interpersonal work but also integrated and continuous management of adherence and stock control of drugs. Recently, their jobs have continued to increased due to the addition of interpersonal work. Stress due to the increase in work load and limitation of time to execute the jobs associated with the expansion of the pharmacists’ role was included in the third factor. Furthermore, precision of operations is also required of pharmacists to secure safe drug therapy for patients [13, 14], and the difficulty in executing a huge amount of work while ensuring the accuracy is considered to be another source of stress included in the third factor.

The fourth and fifth factors were stress caused by the increase in the opportunities of directly dealing with patients, their families, and workers of other professions associated with the involvement of pharmacists in home care. The fourth factor “communication with patients and families” consisted of items including stress related to the handling of patients and their families that are difficult to cope with. According to the survey by Nazish et al. [26], there were cases in which physicians and nurses suffered verbal and physical abuse by patients and their families and considered abandoning their jobs due to the consequent mental distress. In addition, Nakajima et al. [13] reported that occupational stress of pharmacists is also caused by abusive language of patients and coping with uncooperative patients. Similar results were obtained in this study concerning the fourth factor. There is concern that pharmacists are vulnerable to the patients’ rejection of support [27], and pharmacists appear to be stressed by the dilemma caused by the patients’ negative responses despite their sincere effort to provide care.

The fifth factor “communication with other professions” included stress items such as the role of pharmacists not being understood by workers of other professions and lack of cooperation with other professions. It has already been reported that pharmacists’ prescription questions and proposals are not accepted by physicians and that it causes a lack of teamwork and stress in interprofessional work. [28] Concerning an overseas study in physicians, also, Khezar et al. [29] showed that physicians are positive about the pharmacists’ roles and have high expectations but simultaneously that physician–pharmacist communication is deficient. Both in Japan and abroad, the difficulty in physician–pharmacist cooperation is considered to be causing stress in pharmacists. In addition, a study in nurses [30] suggested that a physician-dominant superior–inferior relationship persists in healthcare settings in Japan and that there are times when physicians would not listen to other professionals including nurses and when nurses hesitate to complain out of consideration for physicians’ feelings. In pharmacists, also, stress is considered to be induced by the current situation in which they are conscious about the physician-dominant hierarchy and feel it difficult to express their opinions about patients’ drug therapies.

In working with other professions, pharmacists’ stress is also caused by not being able to obtain understanding about their professional opinions concerning drug therapies, etc. [28] Even if pharmacists propose prescriptions more appropriate for patients by considering the dosage form of the drugs used, cooperation may be hampered by conflict of opinions among healthcare professionals based on differences in their expertise. In addition, there is a report that pharmacists’ adherence guidance in home care is misunderstood by other professionals as simple drug delivery due to poor understanding about the contents of pharmacists’ jobs [27]. Such understanding of pharmacists as outsiders of the home care team by other professions is a factor that prevents the participation of pharmacists in home care. The dilemma of the pharmacists’ roles not being understood by other professions and consequent insufficiency of their intervention in patients’ drug therapies appeared to be serving as a stressor.

As observed above, the scale consisted of 5 factors, of which the first, second, fourth, and fifth factors were associated with stress related to interpersonal work, and pharmacists are considered to have become burdened with many kinds of stress derived from interpersonal work. As for the correlations among various factors (Table 3, bottom), a strong positive correlation was observed between the fourth and fifth factors. With interpersonal work increasing, communication with other professions was also suggested to add to the stress of pharmacists already feeling stress in communication with patients and families.

Regarding the criterion-related validity of the PSS, when its relationship with ERI, which is a conventional scale, was evaluated, only the correlation coefficient between the total score of the fifth factor “communication with other professions” of PSS and the total score of “overcommitment” of ERI was not significant (Table 4). Since significant positive correlations were observed among the other factors, the criterion-related validity of this scale is considered to be generally confirmed. In addition, no significant correlation was observed between the total score of the 51 items of the PSS and the total score of the 3 items to evaluate social-desirability, suggesting that this scale is not affected by social-desirability bias.

This study showed that the PSS is constituted as a questionnaire to measure the stress of pharmacists involved in home pharmaceutical care, which primarily consists of interpersonal work. Although questionnaires targeted to pharmacists’ stress caused by objective work have been developed both in Japan and abroad, those targeted to stress derived from interpersonal work have been few, and no questionnaire concerning home care has been developed. The PSS prepared in this study was confirmed to be useful as the first questionnaire to measure stress caused by interpersonal work and home care services.

The pharmacist is reported to be a more stressful profession compared with other professions [16], and pharmacists’ burden is expected to increase further with increases in their workload associated with the development of the community-based integrated care systems. Thus, pharmacists may remain unaware of the stress they foster, leave it unattended, and eventually develop burnout. Since stress shows a wide array of variation associated with lapse of time and change in environment, it is important to measure it regularly. The use of the questionnaire developed in this study as a means for self-monitoring of stress and its timely management is expected to contribute to the prevention of pharmacists from abandoning their job or developing burnout.

Although this study addressed the stress of pharmacists in the recently increasing demand for home care service, the questionnaire developed in this study is also considered to be applicable to investigation of the stress of hospital pharmacists engaged in interpersonal work.

### Limitation of the study

As limitations of this study, it should be noted that pharmacists with a higher interest in stress may have been more likely to respond to the questionnaire than those with less interest, and that the increase in the workload of pharmacists may have made it difficult for them to find enough time to respond to the questionnaire.

However, as the number of data necessary for the highly reliable analysis results is generally five to ten times the number of items [31], and as the validity of application of factor analysis to the data obtained in this study was confirmed by the Kaiser–Mayer–Olkin measure of sampling adequacy, the number of data sufficient for analysis is considered to have been secured.

For the future, it is necessary to assess the present state of pharmacists’ stress in home care services and interpersonal work using this questionnaire and evaluate measures for its management.

## Conclusions

In this study, 51 items of 5 factors, i.e., “difficulty and feeling of incompetence in patient care”, “relationship with superiors and work environment”, “burdens related to work load and work contents”, “communication with patients and families”, and “communication with other professions” were extracted as stressors of pharmacists in home care services. As in a previous study [18], the stress of pharmacists was related to interpersonal work including giving care to patients at home and working with other professions and environmental factors in implementing home care. In addition, the questionnaire developed in this study was shown to have sufficient reliability, internal consistency, and criterion-related validity.

### Supplementary Information

**Additional file 1.** Japanese version of effort–reward imbalance model (ERI) questionnaire. Questionnaire mailed to surveyed pharmacies.

**Additional file 2.** Pharmacist’s Stress Scale for Home Care (PSS). Final stress scale.

## Data Availability

The data collection tools are attached as an additional supporting file within the text.
