# Supplementary material for: Development of the Pharmacist’s Stress Scale for Home Care (PSS) and evaluation of its reliability and validity
Source: J Pharm Policy Pract. 2023 Dec 28;16:170. doi: 10.1186/s40545-023-00610-8 (PMC10753843; doi:10.1186/s40545-023-00610-8)
Supplement: Supplementary file 1 — Additional file 1. Japanese version of effort–reward imbalance model (ERI) questionnaire. Questionnaire mailed to surveyed pharmacies. [file 40545_2023_610_MOESM1_ESM.pdf]

Japanese version of effort-reward imbalance model (ERI) questionnaire

① Check the box that best fits your situation.

		Not true	True but not bothered at all	True and somewhat bothered	True and considerably bothered	True and extremely bothered
1	I am burdened by work and always pressed for time	☐	☐	☐	☐	☐
2	My work is often interrupted by distractions	☐	☐	☐	☐	☐
3	My work involves heavy responsibility	☐	☐	☐	☐	☐
4	I often have to work overtime	☐	☐	☐	☐	☐
5	My job is physically demanding	☐	☐	☐	☐	☐
6	My workload has increased gradually over the past few years	☐	☐	☐	☐	☐
7	I am not treated fairly at my workplace	☐	☐	☐	☐	☐
8	I have little hope of being promoted	☐	☐	☐	☐	☐
9	I am experiencing undesirable changes at my workplace or expecting such changes for the future.	☐	☐	☐	☐	☐
10	I have a fear of losing my job	☐	☐	☐	☐	☐

② Check the box that best fits your situation.

Choices are changed hereafter.

		True	Not true but not bothered at all	Not true and somewhat bothered	Not true and considerably bothered	Not true and considerably bothered
11	I am properly recognized by my superiors	☐	☐	☐	☐	☐
12	I am properly recognized by my colleagues	☐	☐	☐	☐	☐
13	I have enough support from my colleagues in difficult situations	☐	☐	☐	☐	☐
14	My present job sufficiently reflects the level of education and training that I have received	☐	☐	☐	☐	☐
15	Considering all my efforts and achievements, I am properly recognized as a professional and a person	☐	☐	☐	☐	☐
16	Considering all my efforts and achievements, the prospects of the future of my job are appropriate	☐	☐	☐	☐	☐
17	Considering all my efforts and achievements, my wage/income is appropriate	☐	☐	☐	☐	☐

③ Check the box that best fits yourself.

Choices are changed hereafter.

		Not true at all	Not true	True	Absolutely true
OC1	I am susceptible to pressure of time	☐	☐	☐	☐
OC2	I start thinking of work as soon as I wake up in the morning	☐	☐	☐	☐
OC3	When I come home, I can immediately relax and forget all about job	☐	☐	☐	☐
OC4	My close acquaintances say that I sacrifice myself too much for my job	☐	☐	☐	☐
OC5	I cannot relieve myself of work and think only about work even after going to bed.	☐	☐	☐	☐
OC6	I cannot sleep at night if I have to postpone what I had to do today until tomorrow.	☐	☐	☐	☐
