# Supplementary material for: Development of the Pharmacist’s Stress Scale for Home Care (PSS) and evaluation of its reliability and validity
Source: J Pharm Policy Pract. 2023 Dec 28;16:170. doi: 10.1186/s40545-023-00610-8 (PMC10753843; doi:10.1186/s40545-023-00610-8)
Supplement: Supplementary file 2 — Additional file 2. Pharmacist’s Stress Scale for Home Care (PSS). Final stress scale. [file 40545_2023_610_MOESM2_ESM.pdf]

Pharmacist's Stress Scale for Home Care（PSS）

	items	I feel no stress	I feel little stress	I occasionally feel stress	I always feel stress	I always feel strong stress
1	I cannot have prospects of patient care as a pharmacist	☐	☐	☐	☐	☐
2	I cannot give satisfactory care to patients	☐	☐	☐	☐	☐
3	I cannot have contact with or talk to patients at leisure	☐	☐	☐	☐	☐
4	My superiors have views different from mine	☐	☐	☐	☐	☐
5	There is too much to do other than pharmacist's work such as office work	☐	☐	☐	☐	☐
6	I cannot secure enough time to rest	☐	☐	☐	☐	☐
7	I cannot agree with the physicians' policies or thoughts	☐	☐	☐	☐	☐
8	The poor hygienic environment of the homes I visit makes me feel disagreeable	☐	☐	☐	☐	☐
9	I cannot intervene in home care as I wish to and exercise my professional skill	☐	☐	☐	☐	☐
10	I cannot give support to suffering patients or families	☐	☐	☐	☐	☐
11	I have to see patients unable to have prospects for their future	☐	☐	☐	☐	☐
12	Patients shout at or talk abusively to me	☐	☐	☐	☐	☐
13	My superiors do not trust me	☐	☐	☐	☐	☐
14	I have no one at my workplace to confide in or consult with	☐	☐	☐	☐	☐
15	I do not have enough time to give satisfactory care	☐	☐	☐	☐	☐
16	I have difficulty in arranging time for home visits	☐	☐	☐	☐	☐
17	I am not sure about what explanation patients and families are given by the physicians about the treatment and prognosis	☐	☐	☐	☐	☐
18	Care that I gave for the good of patients and families is misunderstood by them	☐	☐	☐	☐	☐
19	I am paid inadequately for my drug dispensation workload	☐	☐	☐	☐	☐
20	The work of pharmacists is not understood by other professions	☐	☐	☐	☐	☐
21	I am helpless in the care of terminal patients	☐	☐	☐	☐	☐
22	I am not sure how I should deal with patients not informed of their prognoses	☐	☐	☐	☐	☐
23	Patients order me to do things	☐	☐	☐	☐	☐
24	My superiors respond slowly	☐	☐	☐	☐	☐
25	I am required to respond immediately in emergencies	☐	☐	☐	☐	☐
26	Physicians respond slowly	☐	☐	☐	☐	☐
27	I must give care to patients who change their language and attitude according to the profession of the healthcare worker	☐	☐	☐	☐	☐
28	I am not trusted by patients and families	☐	☐	☐	☐	☐
29	The employer's policy prevents me from doing what I want to do for patients	☐	☐	☐	☐	☐
30	Preparation of papers such as reports complicates my job	☐	☐	☐	☐	☐
31	The services that I can provide with my skills or by my pharmacy are deficient	☐	☐	☐	☐	☐
32	I cannot adequately handle patients' and families' anxiety and wishes	☐	☐	☐	☐	☐
33	Death of patients I am in charge of or I have made friends with	☐	☐	☐	☐	☐

	items	I feel no stress	I feel little stress	I occasionally feel stress	I always feel stress	I always feel strong stress
34	Patients commit harassing and malicious behavior	☐	☐	☐	☐	☐
35	My colleagues and workers of other professions do not help me when I am in trouble	☐	☐	☐	☐	☐
36	Manpower is deficient	☐	☐	☐	☐	☐
37	I have to handle unexpected jobs	☐	☐	☐	☐	☐
38	My work is not understood by physicians	☐	☐	☐	☐	☐
39	I have to take care of patients who change their language and attitude according to the pharmacist providing service	☐	☐	☐	☐	☐
40	My sincere care is not understood by patients and families	☐	☐	☐	☐	☐
41	I am urged by the management to improve work efficiency	☐	☐	☐	☐	☐
42	I feel burdened by the heavy liaison work with other profession	☐	☐	☐	☐	☐
43	I am helpless about the exacerbation of patients' symptoms	☐	☐	☐	☐	☐
44	My superiors do not support me when I am in trouble	☐	☐	☐	☐	☐
45	I have to work with uncooperative staff members	☐	☐	☐	☐	☐
46	I have to work under time pressure	☐	☐	☐	☐	☐
47	I have to deal with patients outside duty hours	☐	☐	☐	☐	☐
48	I have to work with uncooperative physicians	☐	☐	☐	☐	☐
49	I have to give care to patients difficult to have communication with	☐	☐	☐	☐	☐
50	I feel burdened by having to visit homes alone (e.g.: carrying a heavy baggage, fear of entering the house of a man living alone)	☐	☐	☐	☐	☐
51	I cannot give care to all patients equally when I have to take care of many home visits	☐	☐	☐	☐	☐
